# An antireductant approach ameliorates misfolded proinsulin-induced hyperglycemia and glucose intolerance in male Akita mice

**DOI:** 10.1007/s11357-024-01326-6

**Published:** 2024-09-19

**Authors:** Dwight A. L. Mattocks, Naidu B. Ommi, Virginia L. Malloy, Sailendra N. Nichenametla

**Affiliations:** Animal Science Laboratory, Orentreich Foundation for the Advancement of Science Inc., 855, Route 301, Cold Spring-on-Hudson, NY 10516 USA

**Keywords:** Glutathione, Oxidative stress, Oxidative eustress, Mutant Insulin Diabetes of Youth, Type-1b diabetes, Unfolded protein response, Disulfide bonds, Protein misfolding

## Abstract

Protein folding in the endoplasmic reticulum (ER) requires a high ratio of oxidized to reduced glutathione (GSSG/rGSH). Since the GSSG/rGSH depends on total glutathione (tGSH = GSSG + rGSH) levels, we hypothesized that limiting GSH biosynthesis will ameliorate protein misfolding by enhancing the ER oxidative milieu. As a proof-of-concept, we used DL-buthionine-(S,R)-sulfoximine (BSO) to inhibit GSH biosynthesis in Akita mice, which are prone to proinsulin misfolding. We conducted a 2-week intervention to investigate if BSO was safe and a 6-week intervention to find its effect on glucose intolerance. In both cohorts, male heterozygous Akita (AK) and wild-type (WT) mice were continuously administered 15 mM BSO. No adverse effects were observed on body weight, food intake, and water intake in either cohort. Unaltered levels of plasma aspartate and alanine aminotransferases, and cystatin-C, indicate that BSO was safe. BSO-induced decreases in tGSH were tissue-dependent with maximal effects in the kidneys, where it altered the expression of genes associated with GSH biosynthesis, redox status, and proteostasis. BSO treatment decreased random blood glucose levels to 80% and 67% of levels in untreated mice in short-term and long-term cohorts, respectively, and 6-h fasting blood glucose to 82% and 74% ﻿of levels in untreated mice, respectively. BSO also improved glucose tolerance by 37% in AK mice in the long-term cohort, without affecting insulin tolerance. Neither glucose tolerance nor insulin tolerance were affected in WT. Data indicate that BSO might treat misfolded proinsulin-induced glucose intolerance. Future studies should investigate the effect of BSO on proinsulin misfolding and if it improves glucose intolerance in individuals with Mutant Insulin Diabetes of Youth.

**Graphical abstract:**

1) *Male heterozygous C57BL/6-Ins2*^*Akita*^*/J (AK) mice suffer from misfolded proinsulin-induced glucose intolerance*.

(a) Proinsulin misfolding occurs due to a genetic mutation in *Ins2* gene that substitutes Cys with Tyr, (b) Due to heterozygosity, AK mice produce both wild-type and mutated proinsulin, (c) Mutated proinsulin forms aggregates with itself and with the bystander native proinsulin, (d) Proinsulin aggregation results in lower functional insulin, and (e) AK mice suffer from impaired glucose tolerance.

2) *Treating mice with BSO improved glucose tolerance.*

(a) Mice were treated with continuous administration of 15 mM DL -buthionine-(S,R)-sulfoximine (BSO), an inhibitor of glutathione biosynthesis (b), BSO treatment increased the renal mRNA quantity of several genes involved in glutathione biosynthesis, glutathione redox status, and proteostasis, (c) we hypothesize that BSO-induced changes in cellular redox status and gene expression ameliorates proinsulin aggregation and increases the functional insulin levels in β-cells, and (d) BSO treatment significantly improved glucose intolerance in AK mice. Note: AUC - Area under the curve, GCL -γ-g-glutamylcysteine ligase, GS - Gluatthione synthetase.

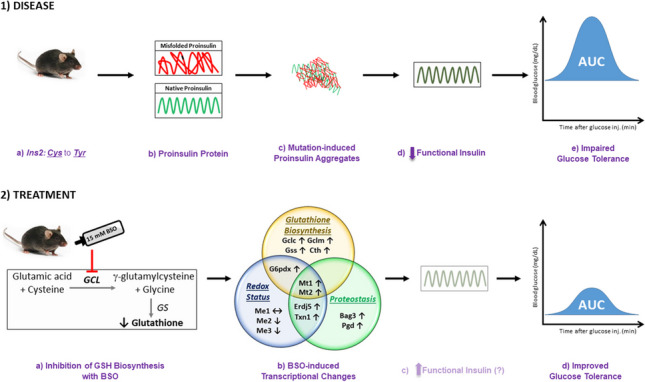

**Supplementary Information:**

The online version contains supplementary material available at 10.1007/s11357-024-01326-6.

## Introduction

Maintaining optimal concentrations of proteins, their structure, and function is essential for normal cellular physiology. Cells employ proteostasis, a complex network of signaling mechanisms and pathways, to ensure proper folding, prevent aggregation, and degrade aggregated proteins. Impaired proteostasis of a specific protein can cause either loss or gain of function, leading to diseases. While widely studied in the context of neurodegenerative diseases, such as Alzheimer’s disease, Parkinson’s disease, and Lou Gehrig’s disease, many non-neurodegenerative diseases, such as cystic fibrosis, retinitis pigmentosa, and Goucher’s disease, also occur due to protein misfolding. Protein misfolding and aggregation also contribute to age-related systemic diseases, including type 2 diabetes, cancers, and chronic inflammation [1–4]. Interventions that prevent protein misfolding and aggregation could increase both healthspan and lifespan. Currently, most therapeutic approaches are directed toward correcting protein quality control systems, including chaperones involved in unfolded protein response, autophagy, and proteasome systems [5–8]. However, physicochemical characteristics, such as pH, cellular concentrations of heavy metals, and redox status of the internal milieu of cells and sub-cellular organelles, are also crucial in the protein folding process. These could be potential targets to treat protein misfolding diseases but are relatively less explored.

About one-third of newly synthesized proteins are misfolded during protein synthesis and degraded immediately to prevent aggregation [9]. Cells can mitigate protein misfolding through the unfolded protein response. However, it is usually transient and initiates cell death if misfolding is not resolved, resulting in the loss of function of non-proliferative tissues [10]. Mitigating the number of misfolded proteins by increasing the fidelity of protein folding machinery might have therapeutic implications for multiple diseases. Disulfide bonds between the constituent cysteine residues of proteins are important in conferring native structure to proteins. Although disulfide bonds can form spontaneously in an oxidizing milieu, many enzymes including protein disulfide isomerase (PDI) and endoplasmic reticulum oxidoreductase (Ero) are essential to ensure bonding between correct cysteine residues, i.e., the formation of native disulfide bonds, and to isomerize non-native disulfide bonds to native disulfides. Both these enzymes act in concert by abstracting electrons from cysteine thiols and are highly reliant on the oxidative environment in the endoplasmic reticulum (ER). The ER provides a relatively higher oxidative environment than the cytosol by preferentially importing oxidized glutathione (GSSG) over reduced glutathione (rGSH); the ratio of GSSG to rGSH (GSSG:rGSH) is much higher in the ER than in the cytoplasm [11]. The higher oxidative milieu ensures a constant flux of oxidizing equivalents required for disulfide bond formation and optimal activity of associated enzymes. Overall, the GSSG:rGSH ratio in the ER and the activities of enzymes involved in protein folding are critical to prevent protein misfolding.

Protein misfolding and aggregation also occurs due to high cellular concentrations of heavy metals. Heavy metals can directly interact with client proteins and promote misfolding or interact with chaperones and prevent them from conferring native structure to client proteins [12–14]. Metallothioneins (MTs), a class of proteins that are well-studied for preventing heavy metal toxicity and mitigating oxidative stress, indirectly prevent such misfolding by sequestrating heavy metals [15, 16]. Notably, MTs are expressed at higher levels in long-lived species and are also shown to be involved in neurodegenerative disorders [17–19]. Transgenic models of *C. elegans* show that increased expression of MTs was associated with decreased amyloid-β and α-synuclein toxicity [20]. The protein expression and functionality of MTs are dependent on cellular redox status, i.e., GSSG/rGSH [21–23]. Thus, increasing GSSG/rGSH might also ameliorate protein misfolding by inducing MTs.

A mild increase in the cellular oxidized environment with beneficial consequences is termed “oxidative eustress” [24]. Studies from our laboratory and others' demonstrate that dietary interventions can induce oxidative eustress [25, 26]. Biosynthesis of GSH, the most abundant small molecule antioxidant, requires the sulfur amino acids, methionine and cysteine. Restricting the dietary intake of these sulfur amino acids not only lowered hepatic total GSH (tGSH = rGSH + GSSG) concentration but also increased GSSG:rGSH, PDI, and ERO1-α, all of which can augment ER protein folding capacity [25, 27, 28]. Likewise, the pharmacological depletion of tGSH increased GSSG:rGSH in animal models [29]. In addition to increasing the GSSG:rGSH, tGSH depletion also increases autophagy, which can eliminate aggregated proteins and increase PDI activity that might further enhance protein folding fidelity [30–32]. Thus, decreasing tGSH levels either by dietary or pharmaceutical means might ameliorate protein-misfolding diseases through multiple mechanisms.

Disulfide bonds play a critical role in insulin biology. Insulin secretion, folding, trafficking, multimerization, and receptor binding are affected by disulfide bond formation [33, 34]. Three disulfide bonds are involved in the formation of native insulin, and one of them occurs between the cysteine residue in the A-chain (A7) and another cysteine residue in the B-chain (B7). A spontaneous mutation in Akita mice substitutes the A7 cysteine with tyrosine, which leaves the B7 cysteine unpaired. The unpaired B7 cysteine forms interloping disulfide bonds with cysteine residues involved in the other two disulfide bonds, A20-B19 and A6-A11. The promiscuous disulfide bonds can occur within the mutant proinsulin or between a mutant proinsulin and a wild-type proinsulin, thus affecting the folding of wild-type proinsulin molecules as well. In heterozygous Akita mice (AK), this process aggravates proinsulin misfolding and results in the aggregation of wild-type proinsulin, which manifests as a progressive type-1 diabetes, termed type-1b diabetes [35]. Type-1b diabetes also occurs in humans, and 26 distinct mutations have been identified to induce misfolding of wild-type proinsulin [36, 37]. Type-1b diabetes in humans caused by proinsulin mutations is termed Mutant Insulin Diabetes of Youth, whose molecular pathology is similar to that of Akita mice. An extremely important but unexplored avenue for treatment is preventing the involvement of non-mutant proinsulin from forming aggregates.

We hypothesized that “inducing oxidative eustress ameliorates diseases associated with protein misfolding.” As a proof of concept, we investigated the effect of mild pharmacological depletion of tGSH on glucose homeostasis in AK mice. In the first cohort, a short-term study, DL-buthionine-(S,R)-sulfoximine (BSO), an inhibitor of GSH biosynthesis, was continuously administered to wild-type (WT) and AK mice in drinking water for 2 weeks, and its effect on morphometry, tGSH, 6-h fasting, and non-fasting glucose (random glucose) was quantified. In the second cohort, a long-term study, WT and littermate male AK mice were bred in-house, and BSO was administered in drinking water for 6 weeks. In addition to the phenotypes quantified in the short-term study, glucose tolerance and insulin tolerance tests were conducted. Data indicate that BSO administration ameliorates glucose intolerance in AK mice.

## Methods

### Animal breeding and genotyping

All animal procedures were reviewed and approved by the Institutional Animal Care and Use Committee of the Orentreich Foundation for the Advancement of Science, Inc. and conducted following NIH guidelines for animal care and use. For the short-term cohort, male AK (C57BL/6-Ins2^Akita^/J, strain No. 003548) and non-littermate male WT (C57BL/6J, Stock No. 000664) mice were purchased from The Jackson Laboratory (Bar Harbor, ME). For the long-term cohort, mice were obtained by crossing a male AK founder with female WT founders in our animal facility. Breeding was continued until the required numbers of male AK and WT littermate pups were obtained. DNA from the tail tips of pups was isolated with DNeasy Blood and Tissue Kit (Qiagen, Germantown, MD, Catalog No. 69504). Genotyping was performed using the protocol, Restriction Enzyme Digest Assay-Ins2^Akita^, the detailed methods of which are available elsewhere [38]. Briefly, after a touchdown PCR, DNA was digested with the restriction enzyme Fnu4HI (New England Biolabs, Ipswich, MA, Catalog No. R0178S). Genotypes were determined based on the number and size of DNA fragments obtained after 2% agarose gel electrophoresis (WT-140 base pairs only; heterozygotes—140 base pair and 280 base pair).

### Animal experiments

In the short-term intervention (2 weeks), 7-week-old mice were divided into four groups of six each: WT mice treated with water [WT_Wtr_] and BSO [WT_BSO_] and AK mice treated with water [AK_Wtr_] and BSO [AK_BSO_]. In the long-term intervention (6 weeks), 9-week-old mice were divided into the same four groups (*n* = 9/group). In both cohorts, BSO groups received 15 mM DL-buthionine-(S,R)-sulfoximine (CAS No. 5072–26-4; Toronto Research Chemicals Inc., Toronto, ON, Catalog No. B690250) in acidified water. Throughout the study, animals were singly housed in a conventional animal facility at 25°C and 50 ± 10% humidity and a 12-h light–dark cycle (7:00 am–7:00 pm). All mice were offered LabDiet 5001 Rodent Chow (PMI Nutrition International, Brentwood, MO) and acidified water with or without BSO ad libitum. Bedding, cages, food, and water were replaced every week. Body weight and food intake were monitored weekly.

At the end of each study, mice were anesthetized with isoflurane, and blood was collected into potassium-EDTA tubes (Sarstedt USA, Newton, NC, Catalog No. K3-EDTA) via the retro-orbital sinus. All mice were immediately euthanized by cervical dislocation. Blood (50 µL) was added to microcentrifuge tubes containing 200 µL of ice-cold 5% meta-phosphoric acid (Sigma-Aldrich, St. Louis, MO, Catalog No. M6288) and snap-frozen in liquid nitrogen. The remainder of the blood was centrifuged at 9000 g for 3 min at 4°C to obtain plasma. The plasma was separated from RBC into new tubes and frozen in liquid nitrogen. Liver and kidneys were collected, weighed, snap-frozen in liquid nitrogen, and stored at − 80°C until analysis. Aliquots of kidneys were stored in RNA*later* at 4°C overnight. After incubation, RNA*later* was discarded and tissues were stored at − 80 °C until used for RNA extraction.

### Glucose homeostasis tests

#### Blood glucose

FreeStyle Lite glucometer (Abbott Laboratories, Abbott Park, IL) was used to quantify random (non-fasting) glucose and 6-h fasting glucose at specific time points during the study. Random glucose levels, i.e., glucose levels without withdrawing food, were measured at approximately 9:00 AM. After withdrawing food at 5:00 AM, 6-h fasting glucose was measured at 11:00 AM. Due to the upper detection limit of the glucometer, all values ≥ 500 mg/dL were considered as 500 mg/dL.

#### Glucose tolerance

An intraperitoneal glucose tolerance test (GTT) was conducted during the last week of intervention. Food was withdrawn at 5:00 AM, and baseline glucose levels were taken at 11:00 AM. Following this, mice were injected with 100 mg/mL d-glucose in water (Sigma-Aldrich, St. Louis, MO, Catalog No. G7021) at a dose of 0.85 g glucose/kg body weight, and the blood glucose levels were measured at 5, 15, 30, 45, 60, 90, 150, 210, and 315 min after the injection. Glucose concentrations greater than 500 mg/dL were considered 500 mg/dL. The area under the curve (AUC) was calculated as the area between the peak of the curve and the average baseline value of the WT_Wtr_ group.

#### Insulin tolerance

The insulin tolerance test (ITT) was performed during the fifth week of the study. Following a 6-h fast starting at 8:00 AM, mice were injected intraperitoneally with 0.1 U/ml Insulin (Humulin R, U-100, Eli Lilly Indianapolis, IN) at a dose of 1.0 U/kg body weight. Glucose levels were monitored until they returned to near baseline values. The area above the curves (AACs) were calculated considering the percent changes from the baseline values in glucose concentrations over time.

### Tissue glutathione

tGSH concentrations were determined as described earlier [6]. Briefly, blood, livers, and kidneys were homogenized in ice-cold 5% metaphosphoric acid, centrifuged at 10,000 g for 15 min at 4°C, and the supernatants were diluted further with GSH Assay Buffer (0.1 M Na_2_HPO_4_, 5 mM EDTA, pH 7.5). tGSH was quantified by an enzymatic recycling method using 5,5-dithiobis-(2-nitrobenzoic acid) as described elsewhere [39]. tGSH concentrations were determined by comparing the rate of color change in unknown samples to known standards.

### mRNA quantity

Total RNA was extracted from the kidneys using TRIzol™ reagent (Invitrogen, Waltham, MA, Catalog No. 15596026). After confirming the RNA quality and concentration using a Nanodrop 2000c Spectrophotometer, 2.5 µg of RNA was treated with DNase-I (Invitrogen, Waltham, MA, Catalog No. 18068015) for 15 min at room temperature. The enzyme was inactivated by adding EDTA (25 mM) for 10 min at 65°C, and RNA was reverse transcribed to cDNA using the High-Capacity cDNA Reverse Transcription Kit with RNase Inhibitor (Applied Biosystems, Waltham, MA, Catalog No. 4374966). cDNA was diluted tenfold with water and quantitative PCR assays were performed in a StepOnePlus real-time PCR system (Applied Biosystems, Waltham, MA) using TaqMan chemistry. All TaqMan assays spanning exon-exon junctions were obtained from ThermoFisher Scientific (Supplementary Table 1). Samples were run in duplicates, and *Ppib* was used as an internal control. Relative gene expression was determined by the ΔΔC_T_ method.

### Triglyceride concentrations

#### Plasma triglycerides

Triglycerides were quantified using a colorimetric assay. Triglyceride stock solution (200 mg/dL) was obtained from Pointe Scientific (Cat No. T7531-STD) and serially diluted with water to make standards. Standards and samples (10 µL) were added to a microplate in duplicate, and 200 µL of Infinity Triglyceride Reagent (ThermoScientific, Catalog No. TR22421) was added per well. The plate was incubated at 37°C for 5 min, and the absorbance was read at 500 nm with a reference wavelength of 660 nm. Triglyceride concentration was determined based on the standard curve.

#### Liver triglycerides

Approximately 50 mg of livers was homogenized in 5 mL of 1 M NaCl on ice in Potter–Elvehjem tubes, and the homogenate was transferred to 50-mL centrifuge tubes. The homogenate was mixed with 10 mL of 2:1 chloroform–methanol solution, vigorously vortexed, and centrifuged for 10 min at 2600 × *g* and room temperature. The lower organic phase was transferred to a clean glass tube. The remnant in the 50-mL tube was mixed with another 5 ml of chloroform–methanol, vortexed, and recentrifuged. Organic phases from both extractions were pooled and dried in an Organomation N-EVAP 112 nitrogen evaporator at 37 °C. Dried lipids were resuspended in 1 ml of 2% Tyloxapol (Sigma-Aldrich, Catalog No. T0307) in chloroform, and the solution was dried again. The final lipid residue was suspended in 1 mL water and the triglyceride concentrations were determined as described for plasma triglycerides.

### Plasma markers of liver and kidney damage

Enzyme-linked immunosorbent assays were used to quantify plasma concentrations of aspartate aminotransferase (AST, Catalog No. ab263882, Abcam, Cambridge, UK), alanine transaminase (ALT, Catalog No. ab282882, Abcam, Cambridge, UK), and cystatin-C (Catalog No. ab201280, Abcam, Cambridge, UK). Plasma insulin and C-peptide concentrations were quantified using C-Peptide (Mouse) ELISA (ALPCO, Salem, NH, Catalog No. 80-CPTMS-E01) and Mouse Ultrasensitive Insulin ELISA (ALPCO, Salem, NH, Catalog No. 80-INSMSU-E01) assay kits, respectively. All assays were performed following the manufacturer’s recommendations. Each sample was run in duplicate and the concentrations were derived based on the absorbance from the standards.

### Statistical analysis

GraphPad Prism 10 (Graph Pad Software, La Jolla, CA) was used to analyze the data. Data were analyzed by two-way ANOVA, considering genotype (WT or AK) and drug (water or BSO) as independent variables. Sidak’s test was used for post-hoc pairwise comparisons, i.e., between WT_Wtr_ and WT_BSO_ or between AK_Wtr_ and AK_BSO_. The overall effects of genotype, drug, interaction between genotype and drug, and treatment duration were represented by P_G_, P_D_, P_G X D_, and P_RX_, respectively. Three-way ANOVA was used to analyze fasting glucose in both cohorts with treatment duration (before and after the intervention), drug, and genotype as independent variables. In one instance, when the mean difference between two groups was large enough to be different but not found statistically different by Sidak’s test, data were analyzed by Student’s two-tailed *t*-test. *P*-values from Student’s *t*-test were represented by “^#^”. *P*-values ≤ 0.05 were considered statistically significant and indicated by asterisks (* < 0.05, ** < 0.01, *** < 0.001, and **** < 0.0001). The sample size for short-term and long-term interventions was six and nine animals per group, respectively. In some analyses, sample sizes were slightly lower than the number of mice used due to the lack of biospecimen volumes for multiple assays. Group averages were expressed as mean ± standard error of the mean.

## Results

### Short-term BSO administration decreases tGSH levels and improves fasting glucose levels

**Fig. 1:**
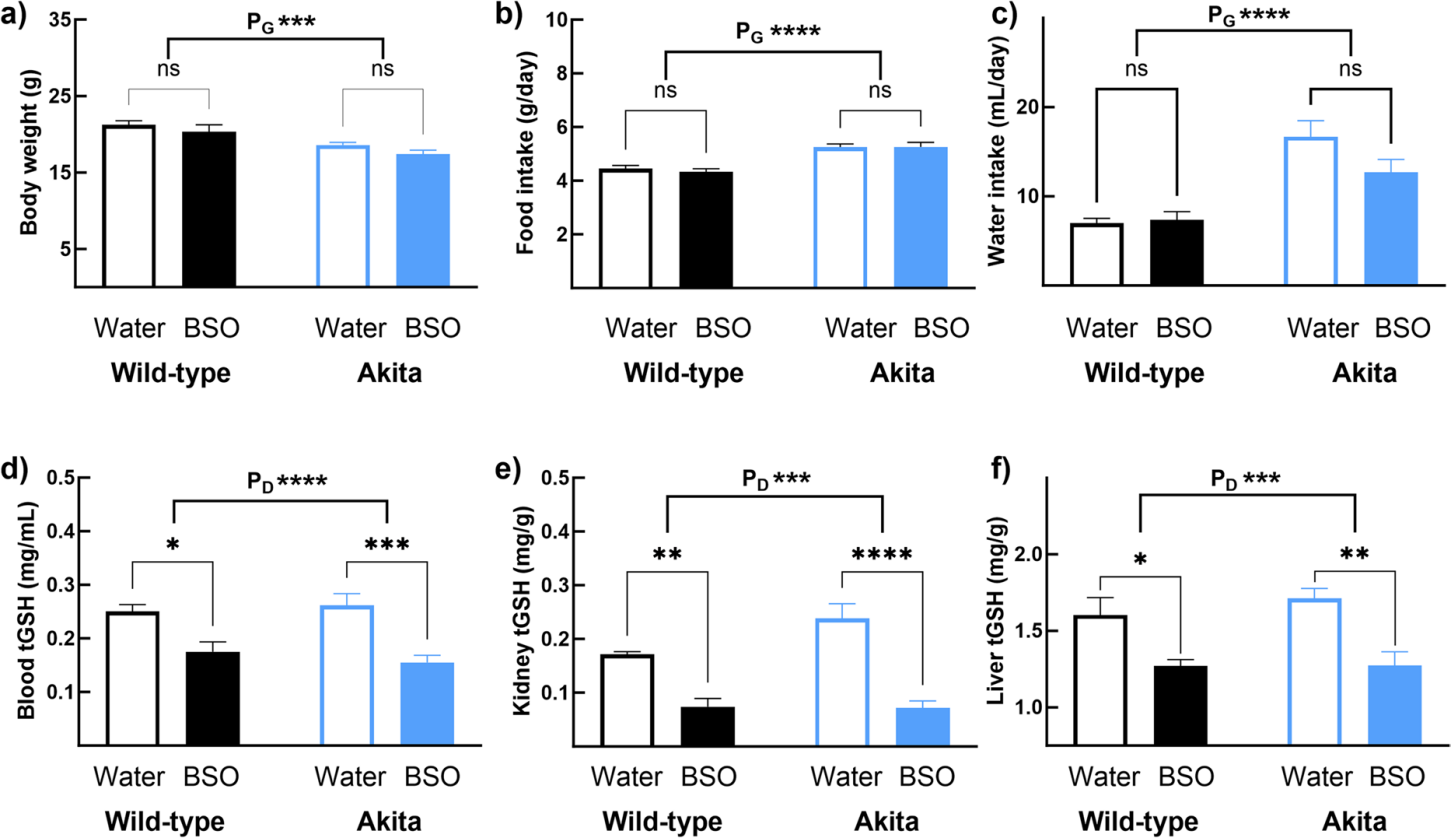
Two-week BSO administration lowers tissue tGSH concentrations without causing adverse effects in Akita mice. Seven-week-old male wild-type (WT) and non-littermate heterozygous Akita mice (AK) mice were given water with or without 15 mM BSO for 2 weeks (experimental groups: WT_Wtr_, WT_BSO_, AK_Wtr_, AK_BSO_; *n* = 6/group). Data were analyzed by two-way ANOVA followed by Sidak’s pairwise comparison test. BSO had no effect on (**a**) body weight, (**b**) food intake, and (**c**) water intake in either WT (WT_Wtr_ = WT_BSO_) or AK (AK_Wtr_ = AK_BSO_). Regardless of the BSO administration, body weight was lower in AK mice than in WT mice (AK_Wtr_ + AK_BSO_ < WT_Wtr_ + WT_BSO_), and food and water intakes were higher (AK_Wtr_ + AK_BSO_ > WT_Wtr_ + WT_BSO_). In both WT and AK mice, BSO lowered tGSH in (**d**) blood, (**e**) kidney, and (**f**) liver. Note: (a) Error bars represent the standard error of the mean. (b) * < 0.05, ** < 0.01, *** < 0.001, **** < 0.00001, and ns, not significant; P_G_—the overall effect of genotype; P_D_—the overall effect of the drug

Since GSH is required for multiple cellular functions, severe depletion would exert adverse effects. Hence, we aimed for only a moderate decrease. Previous studies reported that healthy mice tolerated a 2-week continuous BSO administration up to a concentration of 20 mM [40]. Considering the diabetic status of AK mice and our interest in long-term treatment, we used a concentration of 15 mM, which was well below the tolerated dose. To prevent any effect on growth, we started BSO treatment when body weights plateaued (Supplementary Fig. 1a). Two weeks of continuous administration of 15 mM BSO did not affect body weights in either WT or AK mice (Fig. 1a). However, regardless of the BSO administration, body weights in AK mice were 86% of body weights in WT mice (WT_Wtr_ + WT_BSO_—20.79 ± 0.51 g; AK_Wtr_ + AK_BSO_—18.02 ± 0.33 g, P_G_ < 0.001, Fig. 1a). Similar to its lack of effect on body weight, BSO affected neither food intake nor water intake in WT and AK mice (Figs. 1b and 1c). But the average food intake in AK mice was 116% (WT_Wtr_ + WT_BSO_—4.39 ± 0.08 g/day; AK_Wtr_ + AK_BSO_—5.26 ± 0.10 g/day, P_G_ < 0.0001, Fig. 1b) and water intake was 200% of that in WT mice (WT_Wtr_ + WT_BSO_—7.17 ± 0.15 mL/day; AK_Wtr_ + AK_BSO_—14.70 ± 1.25 mL/day, P_G_ < 0.0001, Fig. 1c). Liver and kidney weights remained unchanged regardless of BSO treatment (Supplementary Figs. 1b and 1c). However, liver weights in AK mice were 115% of those in WT mice (WT_Wtr_ + WT_BSO_—0.86 ± 0.02; AK_Wtr_ + AK_BSO_—0.99 ± 0.03, P_G_ < 0.01, Supplementary Fig. 1b).

tGSH concentrations in the blood, kidney, and liver of WT_BSO_ were 72% (WT_Wtr_—0.25 ± 0.01 mg/mL; WT_BSO_—0.18 ± 0.02 mg/mL, *p* < 0.05), 41% (WT_Wtr_—0.17 ± 0.01 mg/g; WT_BSO_—0.07 ± 0.01 mg/g, and *p* < 0.002), and 79% (WT_Wtr_—1.60 ± 0.11 mg/g; WT_BSO_—1.27 ± 0.04 mg/g, and *p* < 0.02), respectively, of WT_Wtr_ mice (Figs. 1d–1f). A similar effect was found in AK_BSO_ where tGSH in the blood, kidney, and liver was 61% (AK_Wtr_—0.26 ± 0.02 mg/mL; AK_BSO_—0.16 ± 0.01 mg/mL, *p* < 0.001), 29% (AK_Wtr_—0.24 ± 0.03 mg/g; AK_BSO_—0.07 ± 0.01 mg/g, *p* < 0.0001), and 74% (AK_Wtr_—1.71 ± 0.07 mg/g; AK_BSO_—1.27 ± 0.09 mg/g, *p* < 0.01), respectively, of AK_Wtr_ (Figs. 1d–1f). No interaction was observed between BSO and genotype.

**Fig. 2:**
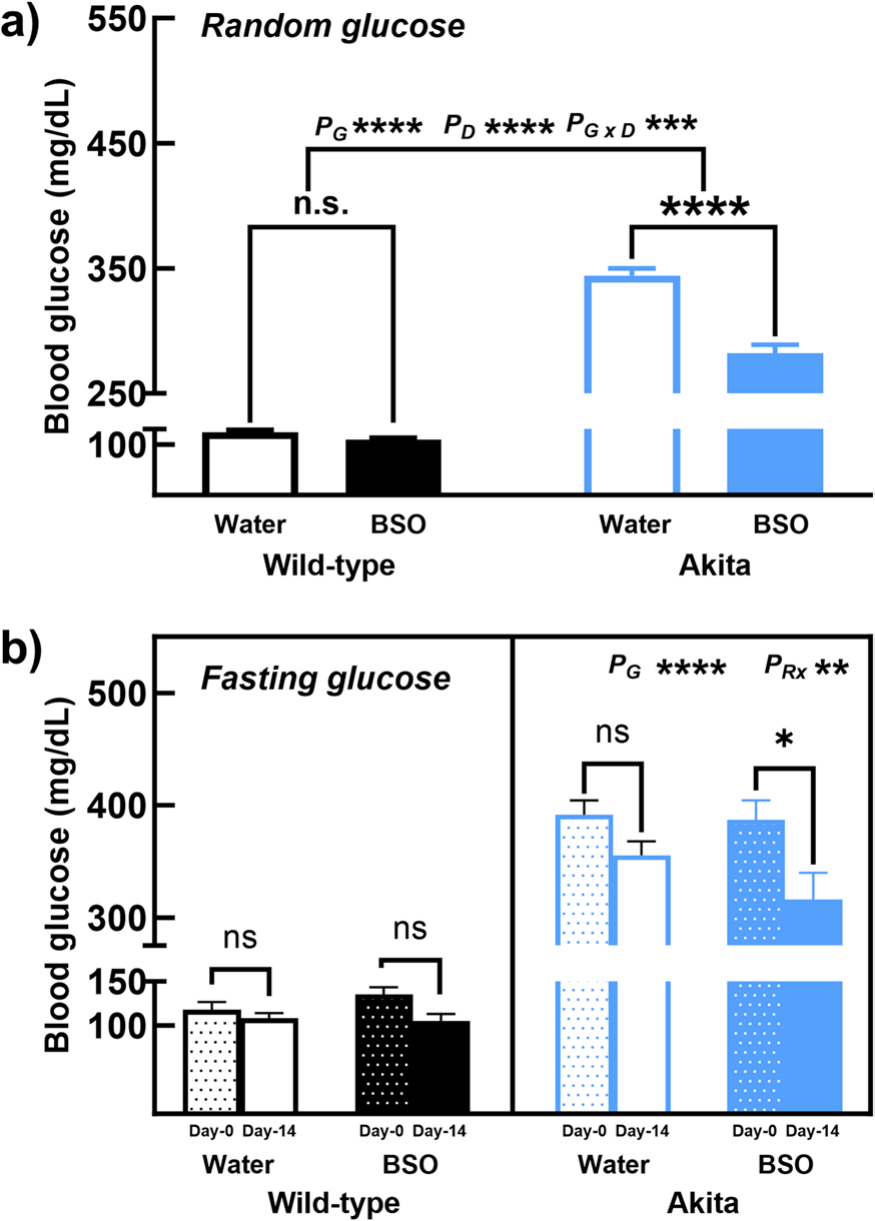
Two-week BSO administration improves random and fasting glucose levels. In the 2-week intervention, BSO decreased (**a**) random glucose levels and (**b**) 6-h fasting glucose levels in AK mice but had no effect in WT mice. Note: (a) Error bars represent the standard error of the mean (b) * < 0.05, ** < 0.01, *** < 0.001, **** < 0.00001, and ns, not significant; P_G_—the overall effect of genotype; P_D_—the overall effect of the drug; P_G X D_—interaction between genotype and drug; and P_Rx_—overall effect of BSO treatment

The average random glucose levels in AK_BSO_ from day 2 through day 13 were 80% of those in AK_Wtr_ (Fig. 2a). The 2-week administration of BSO lowered 6-h fasting glucose in AK_BSO_ to 82% of levels in untreated mice, but had no effect in WT mice (AK_BSO-Day0_—387.17 ± 17.37; AK_BSO-Day14_—316.33 ± 23.53, *p* < 0.05, Fig. 2b). Overall, the 2-week administration of BSO lowered tGSH levels in multiple organs, improved 6-h fasting glucose levels, and random glucose levels without affecting body weights.

### Long-term BSO administration induces tissue-specific changes in glutathione concentration without adverse effects

**Fig. 3:**
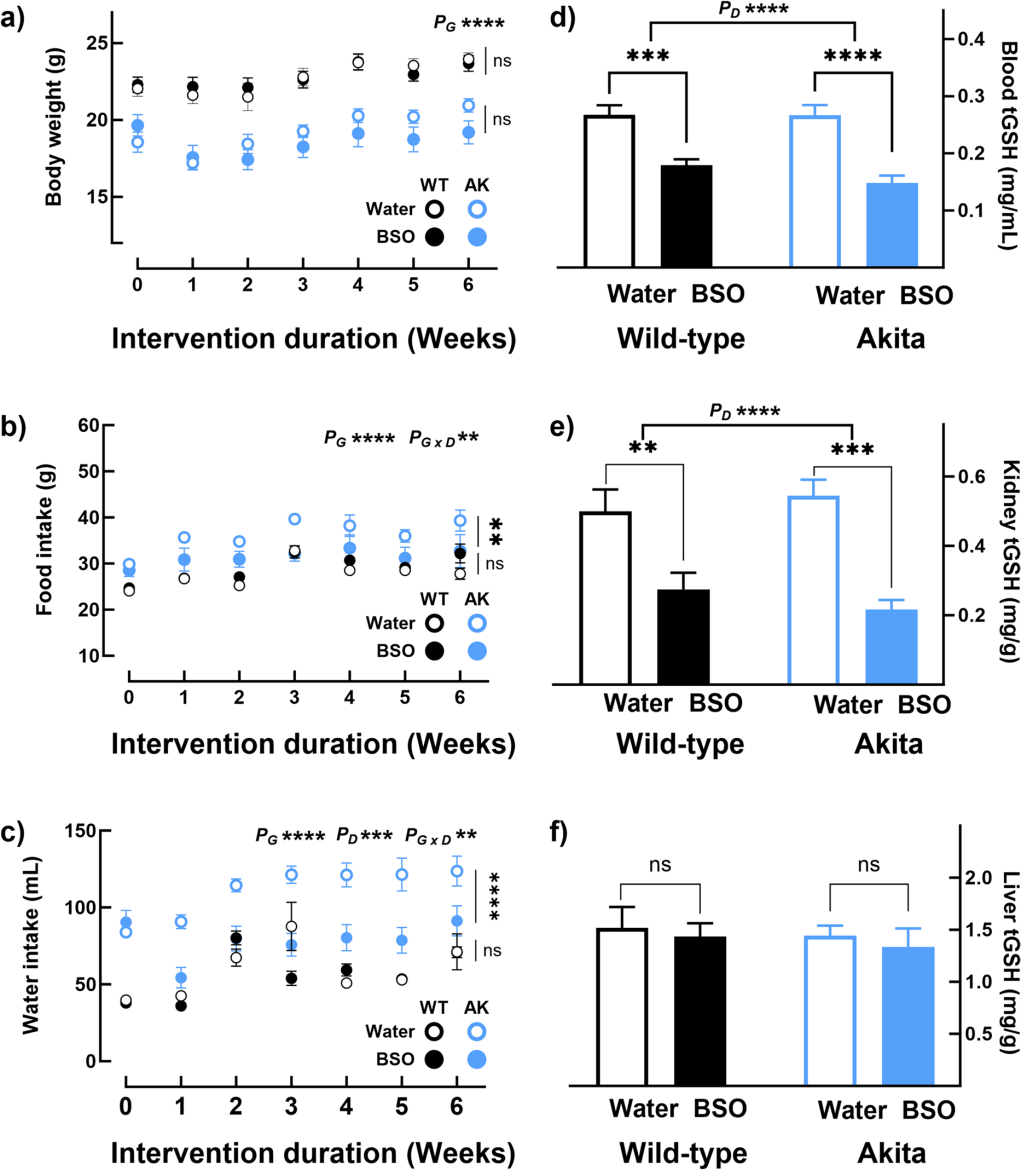
Six-week BSO administration improved food and water intake in Akita mice and lowered tGSH without causing adverse effects. Nine-week-old male AK mice and littermate WT mice were continuously given water with or without 15 mM BSO for 6 weeks (four groups: WT_Wtr_, WT_BSO_, AK_Wtr_, AK_BSO_; *n* = 8–9/group). Data were analyzed by two-way ANOVA followed by Sidak’s pairwise comparison test. BSO had no effect on (**a**) body weight but (**b**) decreased food intake and (**c**) water intake only in AK mice. Regardless of the genotype, BSO exerted similar effects on tissue tGSH, i.e., decreased tGSH levels in (**d**) blood and (**e**) kidney, but (**f**) had no effect in the liver. Note: (a) Error bars represent the standard error of the mean (b)* < 0.05, ** < 0.01, *** < 0.001, **** < 0.00001, and ns, not significant; P_G_—the overall effect of genotype; P_D_—the overall effect of the drug; and P_G X D_—interaction between genotype and drug

To assess if BSO treatment improves glucose tolerance, 15 mM BSO was administered to 9-week-old male AK and WT littermates for 6 weeks. BSO did not affect body weight in WT and AK mice (Fig. 3a). However, the average weekly food and water intakes in AK_BSO_ were 85% (AK_Wtr_—37.27 ± 1.15 g; AK_BSO_—31.88 ± 1.95 g, *p* < 0.01, Fig. 3b) and 66% (AK_Wtr_—115.44 ± 4.17 mL; AK_BSO_—76.73 ± 4.30 mL, *p* < 0.001, Fig. 3c), respectively, of AK_Wtr_. The lowered food and water intakes in AK_BSO_ were closer to the levels observed in WT mice; BSO did not affect food and water intakes in WT mice (Figs. 3b and 3c). Overall, the long-term administration of BSO did not exert adverse effects in either WT or AK mice. However, it appears to have a therapeutic effect on polyphagia and polydipsia in AK mice. Plasma concentrations of the liver damage markers, ALT and AST, were similar in all four groups (Supplementary Figs. 1d and 1e). BSO lowered plasma cystatin-C in WT mice (WT_BSO_—80%, *p* < 0.05, Supplementary Fig. 1f) but did not affect AK mice.

Regardless of the genotype, long-term BSO treatment exerted similar effects on tissue tGSH. Blood tGSH levels in WT_BSO_ were 67% of that in WT_Wtr_ (WT_Wtr_—0.267 ± 0.016 mg/mL; WT_BSO_—0.178 ± 0.010 mg/mL, *p* < 0.001, Fig. 3d) and in AK_BSO_ were 55% of AK_Wtr_ (AK_Wtr_—0.266 ± 0.017 mg/mL; AK_BSO_—0.147 ± 0.012 µg/mL, *p* < 0.0001, Fig. 3d). Compared to their respective controls, BSO also lowered kidney tGSH concentration in WT to 55% (WT_Wtr_—0.499 ± 0.063 mg/g; WT_BSO_—0.274 ± 0.048 mg/g, *p* < 0.01, Fig. 3e) and in AK to 40% (AK_Wtr_—0.544 ± 0.046 mg/g; AK_BSO_—0.216 ± 0.027 mg/g,* p* < 0.001, Fig. 3e). Of note, the extent of the decrease in kidneys was greater than it was in the blood (WT_KID-BSO_—55% of WT_KID-Wtr_; WT_BLD-BSO_—66% of WT_BLD-Wtr_; AK_KID-BSO_—40% of AK_KID-Wtr_; AK_BLD-BSO_—55% of AK_BLD-Wtr_). Contrary to our expectation, liver tGSH levels were unaltered in both WT and AK mice (Fig. 3f). In the short-term cohort, although BSO lowered liver tGSH levels, the effect was mild compared to its effect on blood and kidneys (Figs. 1d–1f).

### Long-term BSO administration improves glucose tolerance in Akita mice

**Fig. 4:**
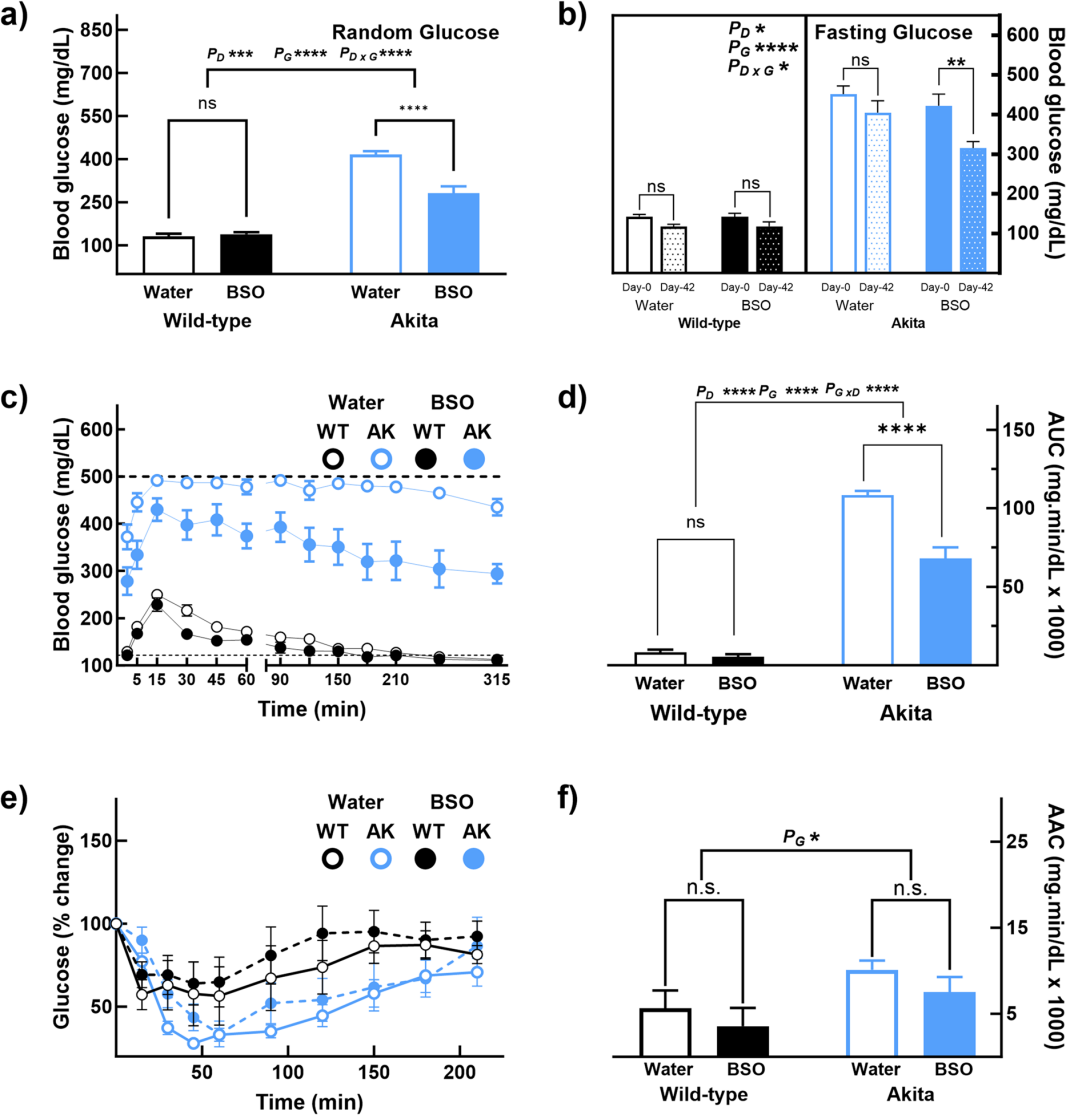
Six-week BSO administration improved glucose homeostasis in AK mice. Six-week BSO treatment (**a**) decreased random glucose levels within 3 days after intervention and (**b**) lowered fasting glucose levels in AK mice but not in WT mice. BSO also (**c**, **d**) improved glucose tolerance in AK mice, while WT mice were unaffected, (**e**, **f**) BSO had no effect on insulin tolerance either in WT or in AK mice. Note: (a) Error bars represent the standard error of mean, (b) * < 0.05, ** < 0.01, *** < 0.001, **** < 0.00001, and ns, not significant; P_G_—the overall effect of genotype; P_D_—the overall effect of the drug; and P_G X D_—interaction between genotype and drug

BSO had a positive effect on glucose homeostasis in AK mice. To find how soon BSO affects blood glucose, we monitored glucose levels every day since starting the intervention until it decreased. A 33% decrease in random glucose levels was seen by the third day of treatment (AK_Wtr_—415 ± 12 mg/dL; AK_BSO_—280 ± 28 mg/dL, *p* < 0.0001, Fig. 4a). The 6-week BSO treatment lowered 6-h fasting glucose in AK to 75% (AK_BSO-Day0_—422.44 ± 29.08 mg/dL; AK_BSO-Day42_—315.38 ± 16.17 mg/dL, *p* < 0.01, Fig. 4b) but had no effect in WT mice. To test if BSO improves glucose homeostasis, glucose and insulin tolerance tests were conducted during the fifth week of treatment and 1 week apart. BSO improved glucose tolerance in AK mice but not in WT mice (Fig. 4c). The area under the curve for AK_BSO_ was 63% of that in AK_Wtr_ (AK_Wtr_—108,318 ± 2728 mg·min/dL; AK_BSO_—67,821 ± 7200 mg·min/dL, *p* < 0.0001, Figs. 4c and 4d); AUCs were similar in WT mice regardless of the BSO administration. On the other hand, BSO did not affect insulin tolerance either in WT or in AK mice (Figs. 4e and 4f). Since triglyceride concentrations can affect glucose tolerance, we quantified hepatic and plasma triglycerides and found that the concentrations were similar in all four experimental groups (Supplementary Figs. 2a and 2b). These data suggest that BSO directly affects glucose tolerance.

Prompted by improved glucose tolerance in AK_BSO_, we quantified insulin and C-peptide in the plasma of 6-h fasted mice. Contrary to our expectation, AK_BSO_ had lower fasting insulin levels than AK_Wtr_ (AK_Wtr_—0.10 ± 0.03 ng/mL; AK_BSO_—0.02 ± 0.006 ng/mL, *p*_2t_ < 0.05), but similar C-peptide levels. Insulin and C-peptide levels were similar in WT_BSO_ and WT_wtr_ (Supplementary Figs. 3a and 3b). Lower fasting insulin levels do not rule out the effect of BSO on improved GTT.

### BSO alters the expression of genes involved in GSH biosynthesis, redox status, and protein homeostasis in kidneys

**Fig. 5:**
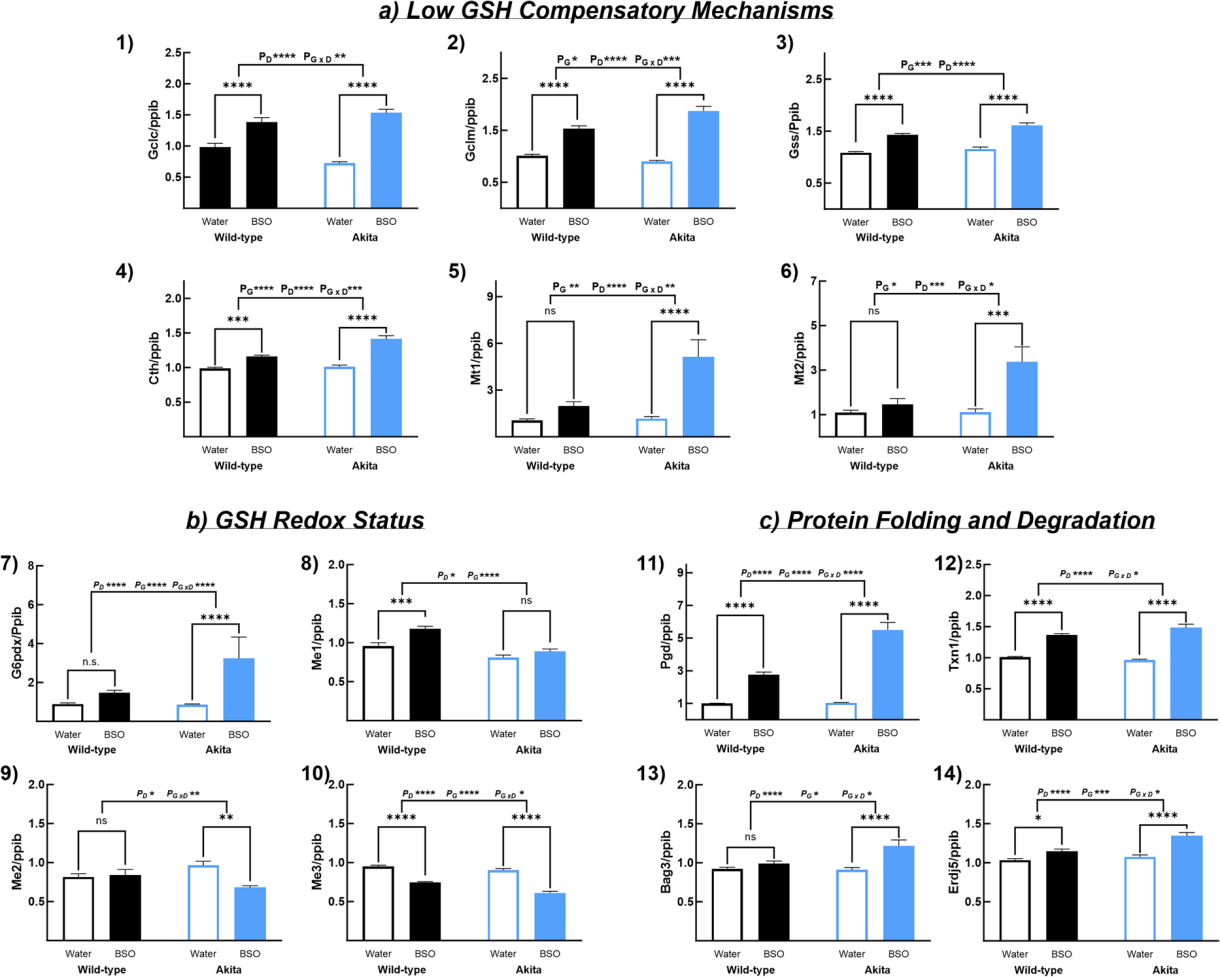
BSO altered the renal mRNA levels of genes associated with GSH biosynthesis, GSH redox status, and protein folding and degradation. In response to BSO treatment, (**a**) mice upregulated several genes involved in GSH biosynthesis (1–6), (**b**) differentially altered the expression of genes associated with cytoplasmic and mitochondrial NADPH-production (7–10), and (**c**) increased the mRNA levels of genes associated with protein folding and degradation (11–14). Note: (a) Error bars represent the standard error of mean, (b) * < 0.05, ** < 0.01, *** < 0.001, **** < 0.00001, and ns, not significant; P_G_—the overall effect of genotype; P_D_—the overall effect of the drug; and P_G X D_—interaction between genotype and drug

To gain mechanistic insights, we quantified mRNA levels of several genes involved in mechanisms that compensate for low tGSH (*Gclc*, *Gclm*, *Gss*, and *Cth*), GSH redox status (*G6pdx*, *Me1*, *Me2*, and *Me3*), and protein folding and degradation (*Pgd*, *Txn1*, *Bag3*, and *Erdj5*), and other genes associated with one or more of these functions (*G6pdx*, *Mt1*, *Mt2*, *Erdj5*, and *Txn1*) in kidneys. BSO increased the mRNA expression of genes associated with GSH biosynthesis (*Gclc, Gclm, Gss*, and *Cth*, Fig. 5a) and those complementing its antioxidant function (*Mt1* and *Mt2*, Fig. 5a). Except for *Gss*, BSO had a greater effect on all these genes in AK mice than in WT mice (P_G X D_ < 0.05). The increase ranged from a mild 117% for *Cth* (WT_BSO_—117% of WT_Wtr_; *p* < 0.0005; AK_BSO_—140% of AK_Wtr_, *p* < 0.0001) to a robust 439% for *Mt1* in AK mice (WT—no change; AK_BSO_—439% of AK_Wtr_, *p* < 0.0001). BSO exerted differential effects on genes associated with GSH redox status. It increased the expression of cytosolic NADPH-generating enzymes *G6pdx* (WT—no change; AK_BSO_—376% of AK_Wtr_, *p* < 0.0001) and *Me1* (WT_BSO_—122% of WT_Wtr_, *p* < 0.0005; AK—no change) but decreased the mitochondrial NADPH-generating enzymes *Me2* (WT—no change; AK_BSO_—70% of AK_Wtr_, *p* < 0.005) and *Me3* (WT_BSO_—78% of WT_Wtr_, *p* < 0.0001; AK_BSO_—67% of AK_Wtr_, *p* < 0.0001, Fig. 5b). Except for *Me1*, for all genes, the effect was greater in AK mice than in WT mice (P_G X D_ < 0.05). In genes associated with protein folding and degradation, BSO had the greatest effect on *Pgd* (WT_BSO_—276% of WT_Wtr_, *p* < 0.0001; AK_BSO_—532% of AK_Wtr_, *p* < 0.0001, Fig. 5c) and the mildest effect on *Erdj5* (WT_BSO_—110% of WT_Wtr_, *p* < 0.05; AK_BSO_—125% of AK_Wtr_, *p* < 0.0001, Fig. 5c).

## Discussion

With oxygen emergence on earth, most life forms have adapted to aerobic life by developing robust enzymatic and non-enzymatic networks to not only effectively handle excess reactive oxygen species, but also to utilize them at low concentrations. Thus, moderate levels of reactive oxygen species and the intracellular oxidative environment became physiologically critical. Recent studies propose that a disproportionate shift in the cellular environment towards a reduced state results in reductive stress, which might lead to diseases [41]. Evidence from clinical studies supports this idea, as excessive supplementation of exogenous antioxidants worsened rather than improving disease severity [42]. On the other hand, empirical data on the potential benefits of mitigating reductive stress or increasing the oxidative milieu is rare. In this study, we provide preliminary evidence for the therapeutic effect of an antireductant approach, i.e., improved glucose tolerance upon a moderate increase in oxidative milieu by limiting the biosynthesis of the most abundant cellular antioxidant, GSH.

We chose the Akita mouse model to test our hypothesis for multiple reasons. Proinsulin misfolding occurs in the ER and is the primary etiology of glucose intolerance in AK mice. Of the three small molecules that determine cellular redox balance, i.e., NADH, NADPH, and GSH, the oxidative environment in the ER is primarily governed by GSH [11, 43]. In addition, the GSSG:rGSH in the ER is closely associated with the thiol-disulfide status of cysteine residues in the ER-resident proteins and their folding status [44]. Modulating GSH levels using BSO is relatively easier than modulating NADH and NADPH. The ultimate readout of the treatment, i.e., blood glucose, is also easy to quantify. However, despite these advantages, this model also poses certain challenges, such as difficulty in accurately quantifying tGSH or GSSG:rGSH in the ER and tedious methods to determine the extent of misfolded proinsulin. Since this is a proof-of-concept study, we limited our investigation to evaluating the effect of BSO on the final readouts, i.e., blood glucose concentrations and glucose tolerance.

Determining the BSO dose for continuous administration was critical in our experimental design. Earlier reports show that of the multiple concentrations used, i.e., 0, 5, 10, 20, and 30 mM, BSO did not affect body weight at 10 mM but slightly decreased it at 20 mM [40]. The dose we selected, 15 mM, seems appropriate, as it did not decrease body weight in either the short-term or long-term cohort (Figs. 1a and 3a). Notably, despite lower food intake, the body weights of AK_BSO_ were not significantly different from that of AK_Wtr_ (Figs. 3a and 3b) after 6-week BSO administration. We speculate that the similar body weights in AK_BSO_ despite lower food intake than in AK_Wtr_ were due to increased food conversion efficiency, probably due to better glucose utilization. Untreated Akita mice cannot properly utilize glucose for tissue or body mass accretion and excrete a significantly higher proportion of it in the urine [45]. Hence, we consider BSO-induced lower food intake as a therapeutic effect on polyphagia but not a toxic effect. To ensure that BSO does not cause toxicity, we quantified the liver damage markers, plasma ALT and plasma AST, and the kidney damage marker, cystatin-C. No increase in plasma ALT and plasma AST either in WT or in AK mice indicates that continuous administration of 15 mM BSO was not toxic (Supplementary Figs. 1d and 1e). To assess for possible kidney damage, we quantified cystatin-C instead of the typical markers such as blood urea nitrogen and creatinine ratio, as it is a better marker of renal homeostasis [46]. BSO did not induce any changes in plasma cystatin-C, indicating that it is non-toxic (Supplementary Fig. 1f). These data strongly suggest that continuous administration of 15 mM BSO is safe in our model.

The lower GTT area under the curve in AK_BSO_ than in AK_Wtr_ provides strong evidence for BSO’s ameliorating effect on hyperglycemia and glucose intolerance. However, it was reported earlier that BSO decreases plasma triglyceride concentrations and that it can affect insulin sensitivity of peripheral tissues in mice and obese individuals [47–49]. Since plasma and liver triglycerides affect glucose tolerance, we wanted to determine if BSO’s effect on glucose tolerance was confounded by altered triglyceride concentrations [50–52]. Unlike in the previous study, BSO did not decrease liver and plasma triglycerides in our study either in WT or in AK mice (Supplementary Figs. 2a and 2b). This differential effect could be attributed to the differences in the dietary composition between the two studies. Elshorbagy et al. used a high-fat diet with 45% Kcal from fat, whereas the fat composition in our diet was approximately 6% Kcal. It is plausible that BSO might affect triglyceride levels depending on dietary fat composition. To find if BSO affects insulin sensitivity in our model, we performed an ITT. The area above the curve from the ITT was similar in all four groups, demonstrating that BSO’s effect on blood glucose was independent of its effect on insulin sensitivity (Figs. 4e and 4f).

AK_BSO_ mice had lower plasma insulin levels despite better glucose tolerance than AK_Wtr_. This discrepancy could be due to multiple reasons. Fasting insulin concentration is a poor indicator of β-cell insulin secretory capacity. Lower plasma insulin levels in AK_BSO_ could be due to lower fasting glucose rather than poor insulin secretion [53]. We fasted all mice before harvesting plasma and tissues to decrease the variability in tGSH concentrations associated with food intake. While the prandial state during GTT was similar to a fed state, plasma insulin was collected during the fasted state. Plasma insulin levels in our study cannot be used to draw conclusions about β-cell insulin secretory capacity. Despite lower plasma insulin than AK_Wtr,_ AK_BSO_ mice might still have more functional insulin in β-cells that would be released upon stimulation with glucose.

Previous studies demonstrate that cellular redox state plays a crucial role in glucose-stimulated insulin secretion [54]. In addition to ATP, glucose metabolism generates reactive oxygen species in mitochondria, which migrate to the cytoplasm and induce the release of insulin granules. An increase in the GSSG/GSH ratio in mouse islets and INS1 cells during this process suggests that an oxidative environment is required for insulin secretion [31]. In addition, oxidizing agents such as diethylmaleate are known to increase insulin secretion by generating hydrogen peroxide [55]. It is possible that the higher oxidative environment in AK_BSO_ augments insulin secretion only upon glucose injection as in GTT or fed states but not in fasted states where plasma glucose would be minimal. To precisely determine whether BSO has any effect on islet insulin quantity and secretion, future studies should quantify the insulin content in the pancreatic islets and monitor the release of fluorescently tagged proinsulin, insulin, or C-peptide proteins in vivo as described earlier [56].

Our hypothesis was based on the ability of BSO to alter the cellular redox state towards a pro-oxidant milieu by decreasing tGSH concentrations and increasing GSSG:rGSH. Due to its high concentration, GSH is a major determinant of the cellular redox state. However, the concentrations and the ratios of GSSG to rGSH are subcellular compartment-specific [57–59]. It is extremely difficult to quantify the concentrations of GSSG and rGSH in the ER, where proinsulin misfolding occurs. Hence, we quantified the mRNA expression of genes that respond to low cellular GSH levels (*Gclc*, *Gclm*, *Gss*, *Cth*, *Mt1*, and *Mt2*), cell compartment-specific redox status (*Me1*, *Me2*, *Me3*, and *G6pdx*), and those involved in protein folding (*Bag3*, *Erdj5*, *Pgd*, and *Txn1*). Since maximal changes in tGSH concentrations occurred in the kidneys, we quantified the expression of these genes in the kidneys. The increased mRNA expression of *Gclc*, *Gclm*, *Gss*, and *Cth* provides strong evidence for low cellular GSH concentrations, as these genes are highly responsive to such conditions. Notably, the *Mt1* and *Mt2* are induced not only due to low GSH levels but also in an oxidative environment, such as increased reactive oxygen species, and are implicated in protein folding [16, 60]. They also increased the β-cell survival when exposed to reactive oxygen species [61]. In addition to *Mt1* and *Mt2*, the higher expression of several other genes involved in protein folding, autophagy, and protein degradation, including *Bag3*, *Erdj5*, *Pgd*, and *Txn1*, suggests that BSO treatment might improve protein folding fidelity [62–67]. A strong increase in *G6pdx*, the major source of cytosolic NADPH required for converting GSSG to rGSH, indicates that upon BSO treatment, cells try to maintain the cytosol in a reduced state. In contrast, a decrease in the mitochondrial NADPH sources, *Me2* and *Me3*, indicates the opposite. The gene expression data suggests that BSO treatment decreases the GSH concentration and exerts differential effects on the redox status of various cell compartments. These changes, combined with increased proteostatic gene expression, might positively affect protein folding and aggregation.

The current study has some limitations. Although our hypothesis was highly mechanistic, i.e., that BSO improves glucose intolerance in male AK mice by ameliorating proinsulin misfolding, owing to technical difficulties, we did not demonstrate such an effect. Hence, this aspect remains inconclusive, and future studies in this direction are highly recommended. Our data on the effect of BSO on liver tGSH is atypical in that it either did not affect or only slightly decreased hepatic tGSH. We speculate that several factors, including the lower BSO dose, route of administration, sensitivity of different tissues to BSO, and diurnal fluctuations, might contribute to this lack of difference. Previous observations show that kidneys are much more sensitive to BSO administered in water than the liver and that kidneys and the liver respond differently to BSO, even with intraperitoneal administration [40, 68]. In addition, it is well known that tGSH concentrations in the liver fluctuate throughout the day, while such changes are minimal in kidneys [69]. Overall, our study provides a novel target, i.e., subcellular redox environment in the context of protein misfolding and aggregation diseases and highlights that antireductant approaches in certain disease conditions could improve health.

## Supplementary Information

Below is the link to the electronic supplementary material.

Supplementary file 1 (JPEG 888 kb)

High resolution image (TIF 2.10 mb)

Supplementary file 2 (PNG 76 kb)

High resolution image (TIF 295 kb)

Supplementary file 3 (PNG 85 kb)

High resolution image (TIF 323 kb)

Supplementary file 4 (DOCX 31 kb)
