# Supplementary figures and images for: An antireductant approach ameliorates misfolded proinsulin-induced hyperglycemia and glucose intolerance in male Akita mice
Source: GeroScience. 2024 Sep 19;47(2):1653–68. doi: 10.1007/s11357-024-01326-6 (PMC11979071; doi:10.1007/s11357-024-01326-6)

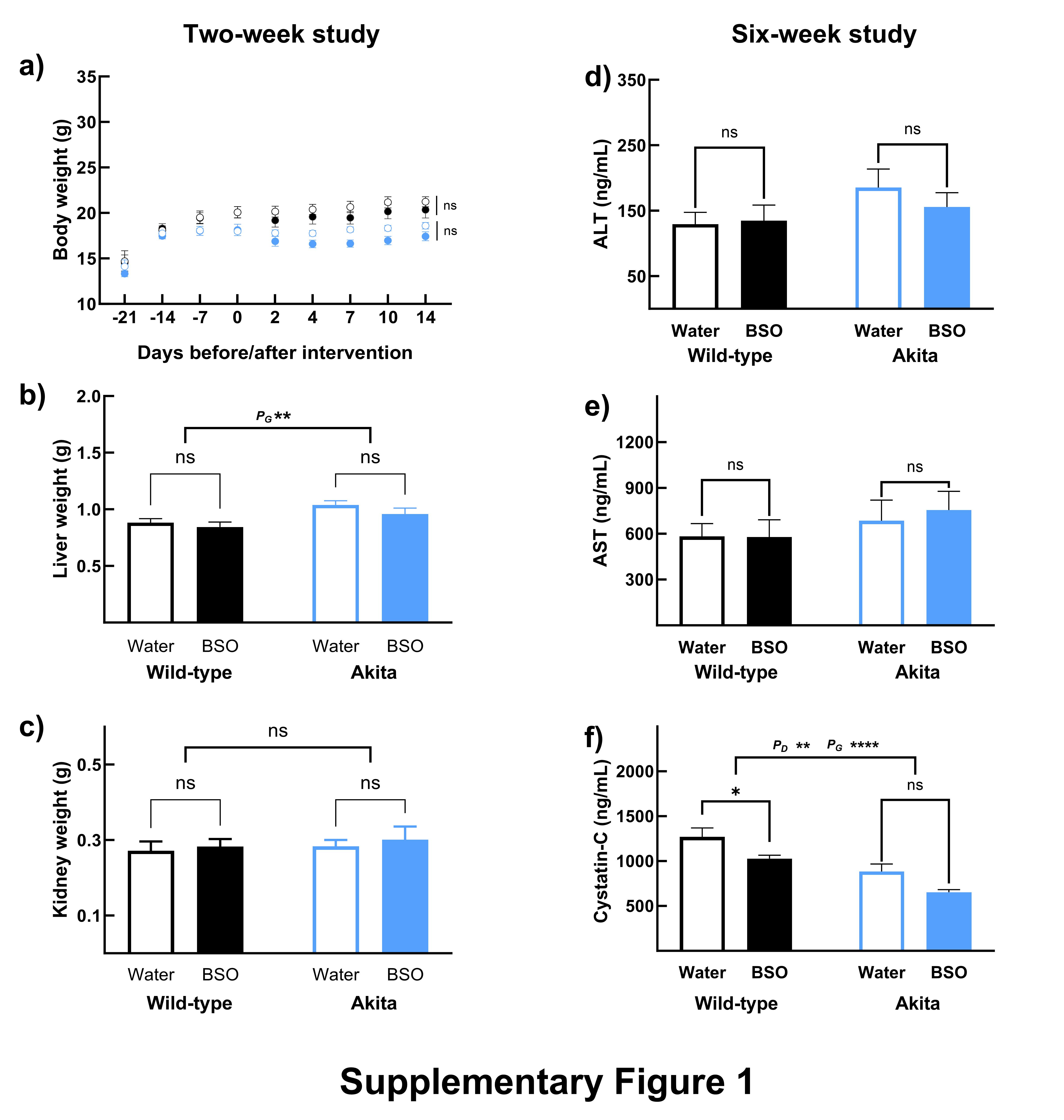

Supplement: Supplementary file 1 — (JPEG 888 kb) [file 11357_2024_1326_Fig6_ESM.jpg]

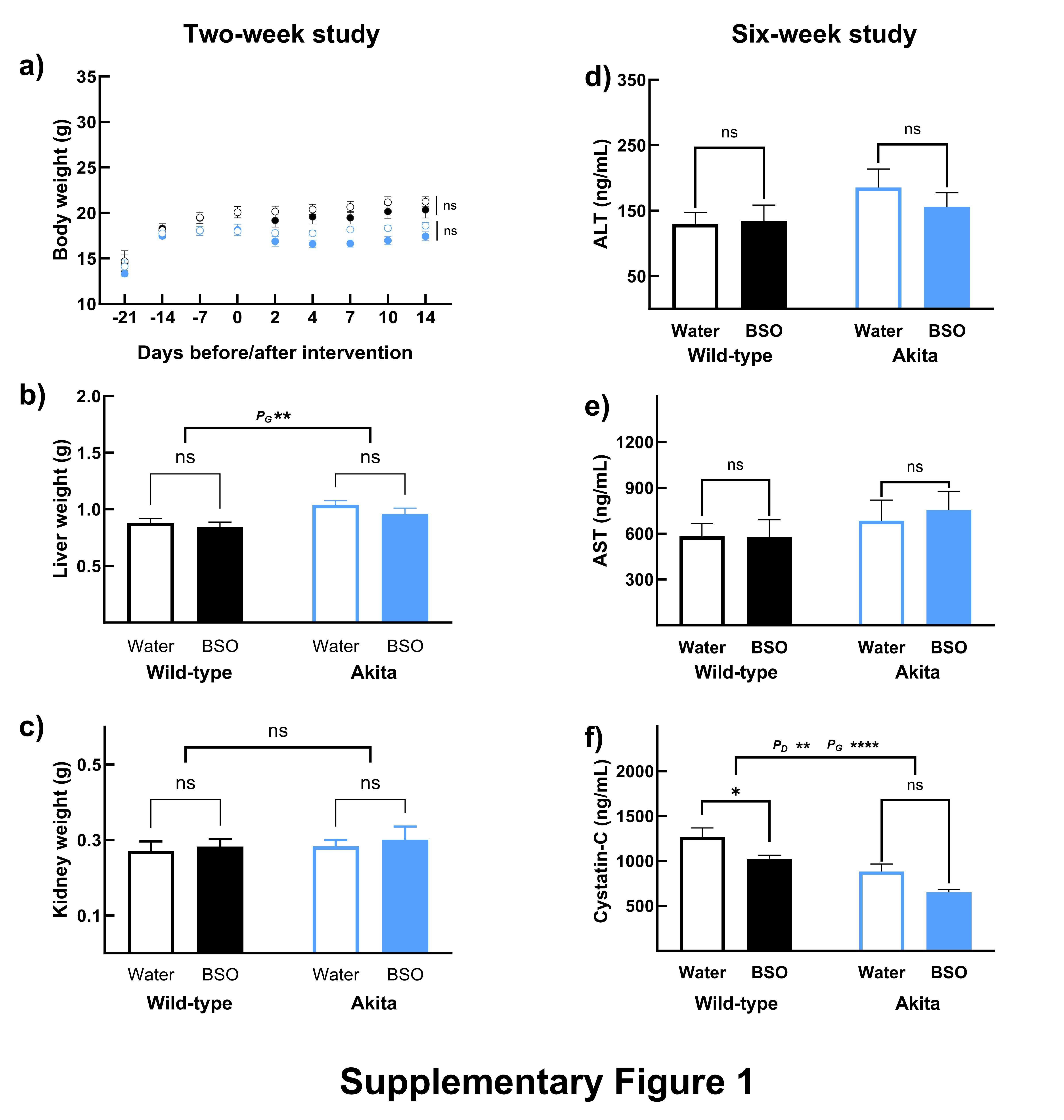

Supplement: Supplementary file 2 — High resolution image (TIF 2.10 mb) [file 11357_2024_1326_MOESM1_ESM.tif]

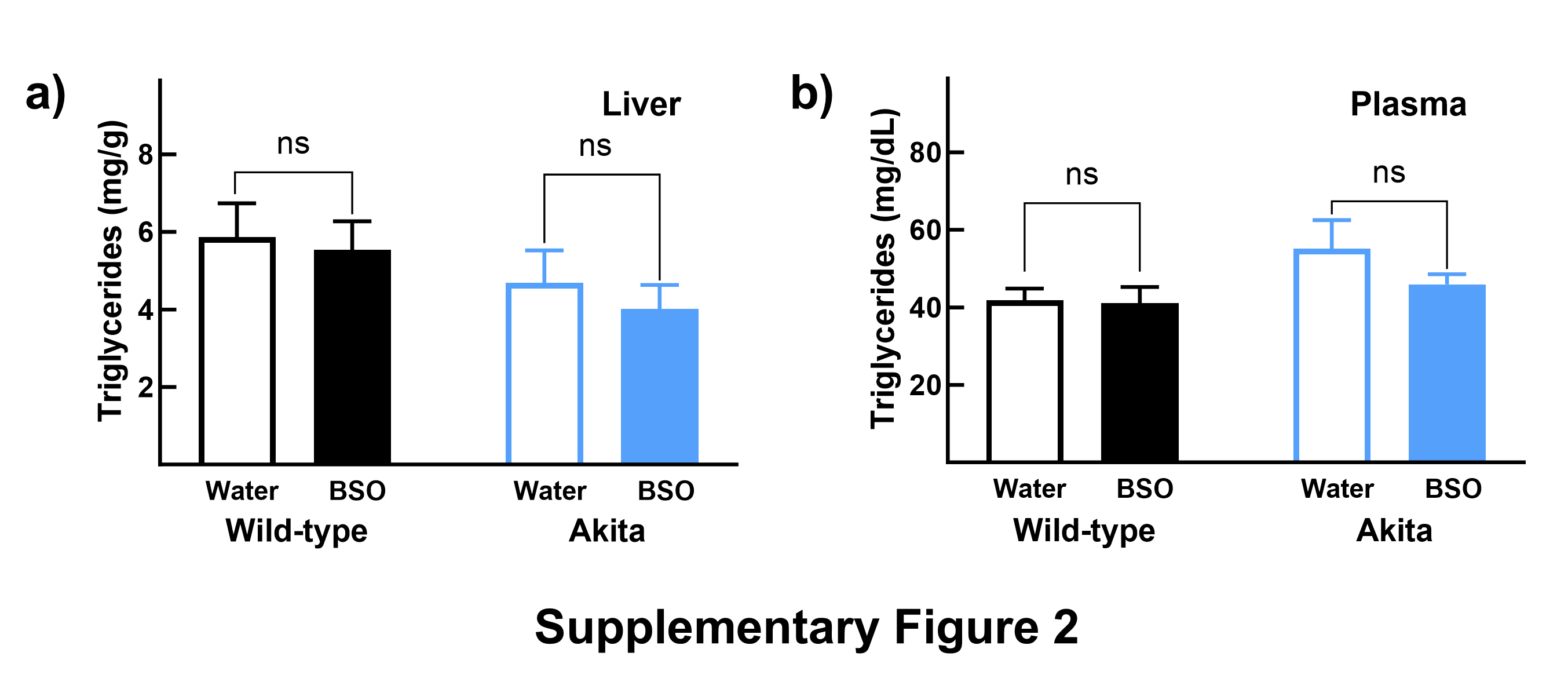

Supplement: Supplementary file 3 — (PNG 76 kb) [file 11357_2024_1326_Fig7_ESM.png]

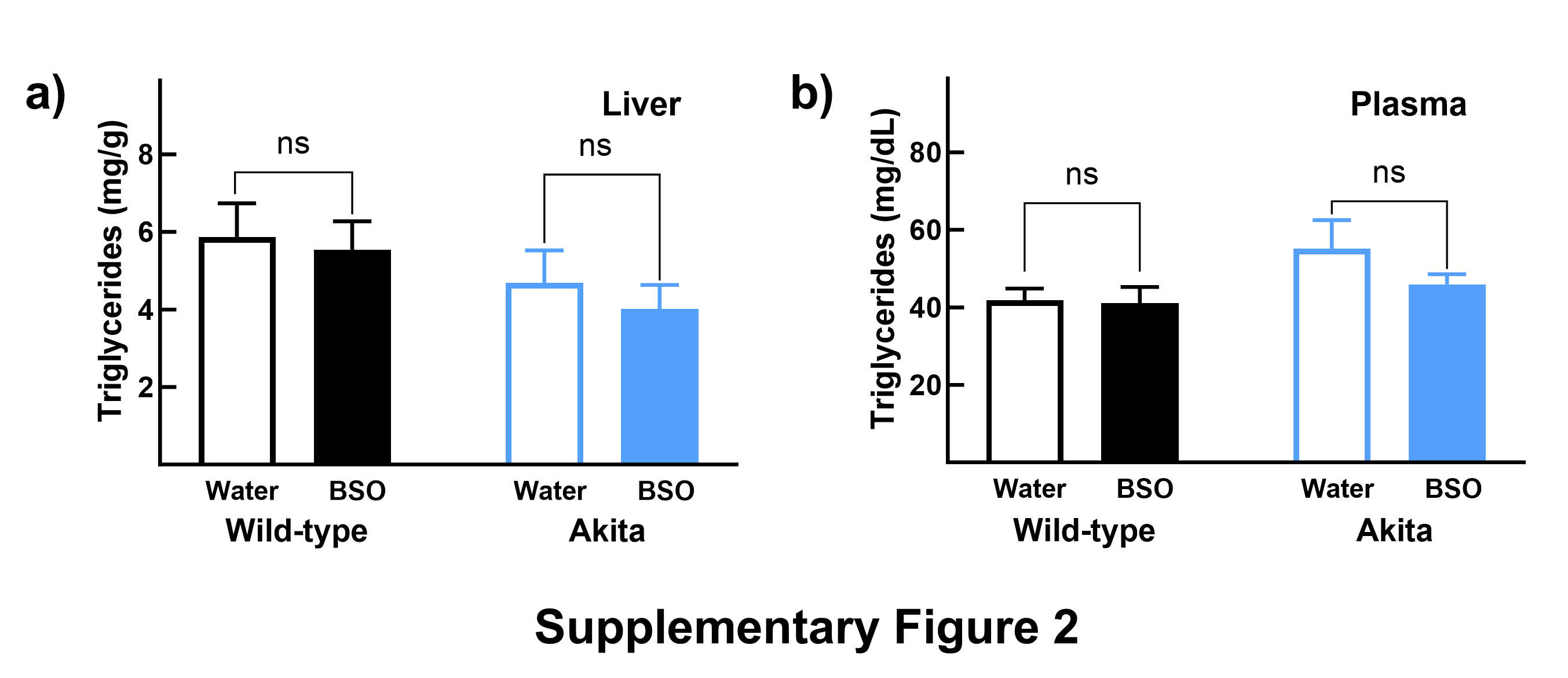

Supplement: Supplementary file 4 — High resolution image (TIF 295 kb) [file 11357_2024_1326_MOESM2_ESM.tif]

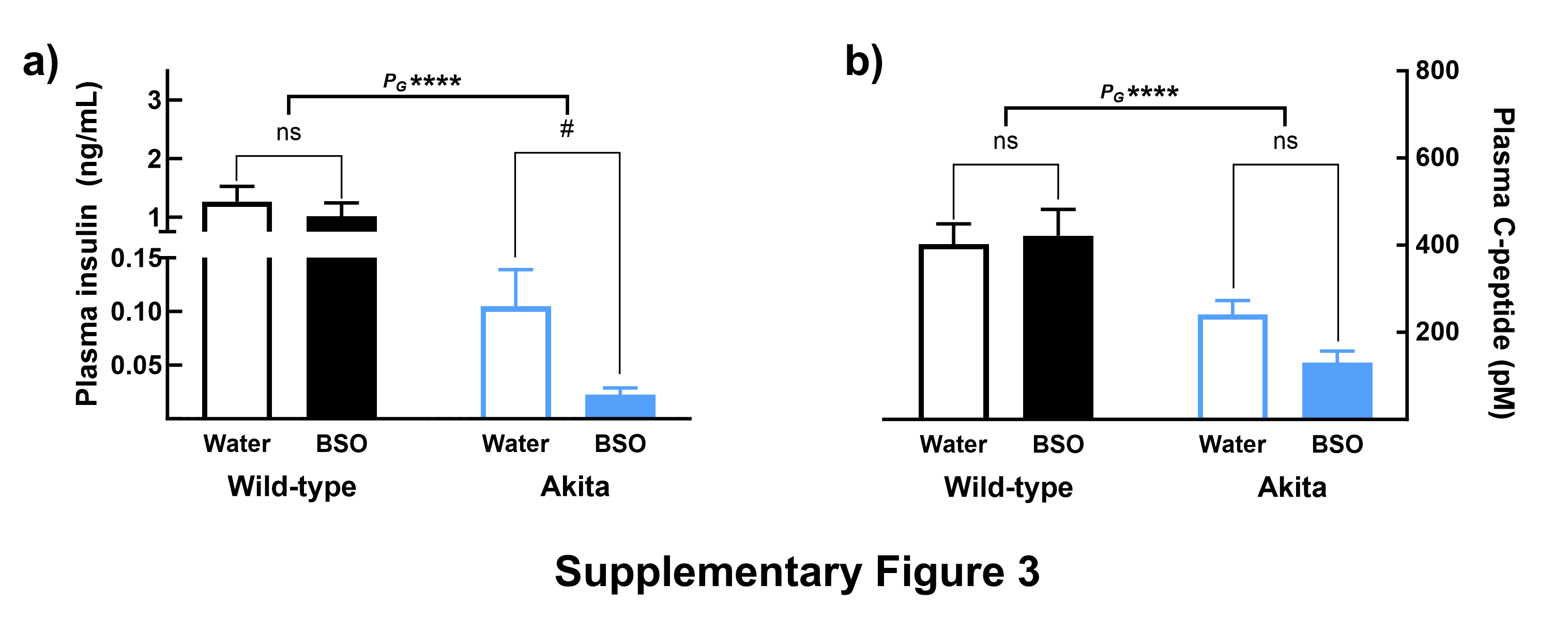

Supplement: Supplementary file 5 — (PNG 85 kb) [file 11357_2024_1326_Fig8_ESM.png]

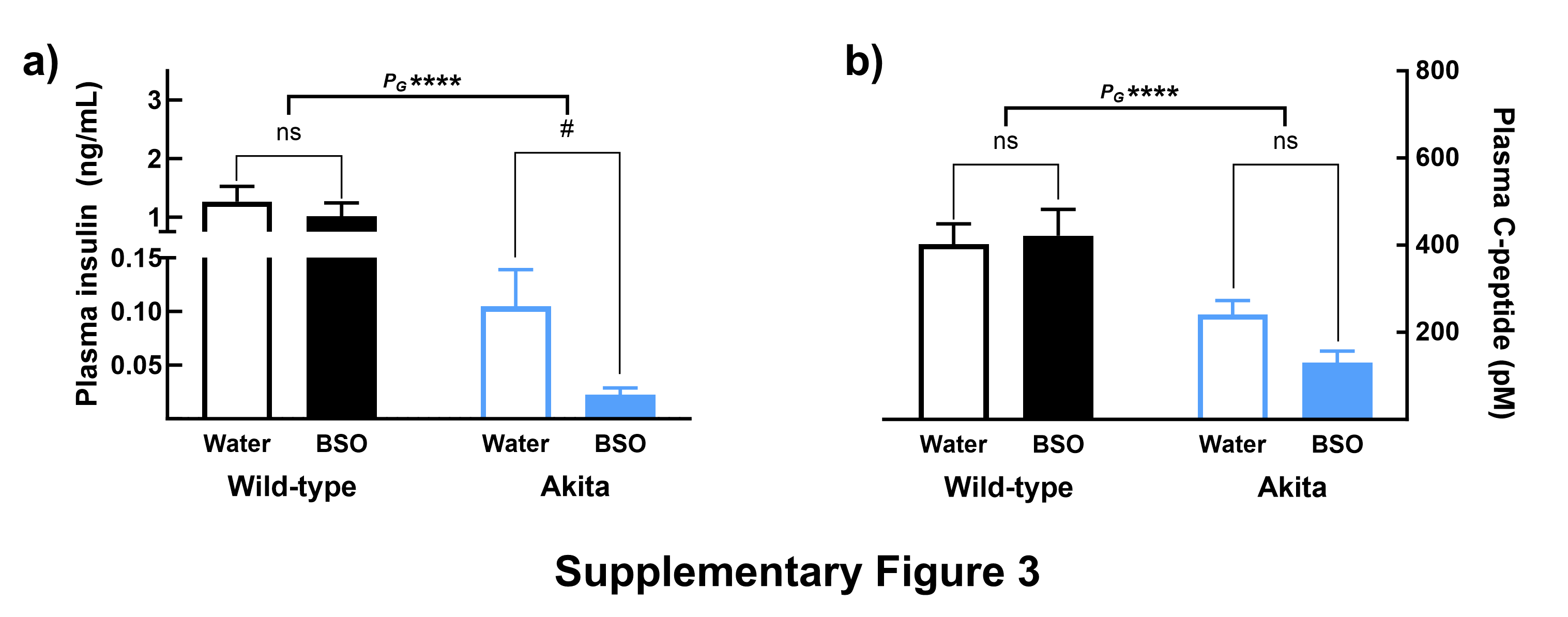

Supplement: Supplementary file 6 — High resolution image (TIF 323 kb) [file 11357_2024_1326_MOESM3_ESM.tif]
