# Supplementary material for: An antireductant approach ameliorates misfolded proinsulin-induced hyperglycemia and glucose intolerance in male Akita mice
Source: GeroScience. 2024 Sep 19;47(2):1653–68. doi: 10.1007/s11357-024-01326-6 (PMC11979071; doi:10.1007/s11357-024-01326-6)
Supplement: Supplementary file 7 — (DOCX 31 kb) [file 11357_2024_1326_MOESM4_ESM.docx]

**Supplementary figure 1.** **BSO administration did not induce adverse effects.**

The two-week BSO administration did not exert adverse effects on (a) body weights, (b) liver weights, and (c) kidney weights in either WT or AK mice. The six-week BSO administration did not increase plasma markers of liver damage, including d) alanine transaminase, e) aspartate transaminase, and kidney damage f) cystatin-C. Note: a) Error bars represent the standard error of mean b) *<0.05, **<0.01, ***<0.001, ****<0.00001, and ns – not significant; P_G_ – the overall effect of genotype; P_D_ – the overall effect of the drug.

**Supplementary figure 2. BSO has no effect on triglyceride concentrations.**

Six-week administration of BSO did not affect triglyceride concentrations in either (a) the liver or (b) the plasma. Note: a) Error bars represent the standard error of mean b) ns – not significant.

**Supplementary figure 3. BSO did not improve fasting plasma insulin.**

BSO had no effect on fasting plasma concentrations of either (a) insulin or (b) c-peptide. An overall genotype effect was observed, where AK mice had lower levels of insulin and c-peptide. Note: a) Error bars represent the standard error of mean b) ****<0.00001 and ns – not significant; # P-value from two-tailed Student’s t-test was < 0.05; P_G_ – the overall effect of genotype.

| **Supplementary Table 1. Technical details of TaqMan assays used in mRNA quantification** | |
| --- | --- |
| **Gene of interest** | **TaqMan assay ID** |
| *Gclc* | *Mm00802658_m1* |
| *Gclm* | *Mm00514997_m1* |
| *Gss* | *Mm01246547_g1* |
| *Cth* | *Mm00461247_m1* |
| *Mt1* | *Mm00496660_g1* |
| *Mt2* | *Mm04207591_g1* |
|  | |
| *G6pdx* | *Mm00656735_g* |
| *Me1* | *Mm07293398_g1* |
| *Me2* | *Mm00521023_m1* |
| *Me3* | *Mm00724881_m1* |
|  | |
| *Pgd* | *Mm00503037_m1* |
| *Txn1* | *Mm01351952_g1* |
| *Bag3* | *Mm00443474_m1* |
| *Erdj5* | *Mm00546464_m1* |
| *Note: All assays were obtained from ThermoFisher Scientific* | |
